# Covariation of Ergot Severity and Alkaloid Content Measured by HPLC and One ELISA Method in Inoculated Winter Rye across Three Isolates and Three European Countries

**DOI:** 10.3390/toxins12110676

**Published:** 2020-10-26

**Authors:** Anna Kodisch, Michael Oberforster, Armin Raditschnig, Bernd Rodemann, Anna Tratwal, Jakub Danielewicz, Marek Korbas, Brigitta Schmiedchen, Jakob Eifler, Andres Gordillo, Dörthe Siekmann, Franz Joachim Fromme, Frederik N. Wuppermann, Franz Wieser, Elisabeth Zechner, Małgorzata Niewińska, Thomas Miedaner

**Affiliations:** 1 State Plant Breeding Institute, University of Hohenheim, 70599 Stuttgart, Germany; kodisch.a@uni-hohenheim.de; 2 Austrian Agency for Health and Food Safety (AGES), Institute for Sustainable Plant Production, 1220 Vienna, Austria; michael.oberforster@ages.at; 3 Austrian Agency for Health and Food Safety (AGES), Institute for Food Safety Linz, 4020 Linz, Austria; armin.raditschnig@ages.at; 4 Federal Research Centre for Cultivated Plants, Julius Kühn-Institute, Institute for Plant Protection in Field Crops and Grassland, 38104 Braunschweig, Germany; bernd.rodemann@julius-kuehn.de; 5 Institute of Plant Protection—National Research Institute, 60-318 Poznań, Poland; a.tratwal@wp.pl (A.T.); j.danielewicz@iorpib.poznan.pl (J.D.); M.Korbas@iorpib.poznan.pl (M.K.); 6 KWS LOCHOW GMBH, 29303 Bergen, Germany; Brigitta.Schmiedchen@kws.com (B.S.); Jakob.Eifler@kws.com (J.E.); Andres.Gordillo@kws.com (A.G.); 7 HYBRO Saatzucht GmbH & Co. KG, 17291 Schenkenberg, Germany; siekmann@hybro.de (D.S.); fromme@hybro.de (F.J.F.); 8 LCTech GmbH, 84419 Obertaufkirchen, Germany; wuppermann@lctech.de; 9 Saatzucht LFS Edelhof, 3910 Zwettl, Austria; f.wieser@saatzucht.edelhof.at (F.W.); e.zechner@saatzucht.edelhof.at (E.Z.); 10 DANKO Hodowla Roslin Sp. z o.o., 64-000 Kościan, Poland; malgorzata.niewinska@danko.pl

**Keywords:** ergot alkaloids, *Claviceps purpurea*, HPLC, mycotoxins, ELISA, rye

## Abstract

Ergot caused by *Claviceps purpurea* is a problem for food and feed security in rye due to the occurrence of toxic ergot alkaloids (EAs). For grain elevators and breeders, a quick, easy-to-handle, and cheap screening assay would have a high economic impact. The study was performed to reveal (1) the covariation of ergot severity (= percentage of sclerotia in harvested grain) and the content of 12 EAs determined by high performance liquid chromatography (HPLC) and (2) the covariation between these traits and results of one commercial enzyme linked immunosorbent assays (ELISA). In total, 372 winter rye samples consisting of a diverse set of genotypes, locations from Germany, Austria, and Poland over two years, and three isolates were analyzed. Ergocornine and α-ergocryptine were detected as major EAs. Ergocristinine occurred as a minor component. *Claviceps* isolates from different countries showed a similar EA spectrum, but different quantities of individual EAs. A moderate, positive covariation between ergot severity and EA content determined by HPLC was observed across two years (*r* = 0.53, *p* < 0.01), but large deviation from the regression was detected. ELISA values did neither correlate with the HPLC results nor with ergot severity. In conclusion, a reliable prediction of the EA content based on ergot severity is, at present, not possible.

## 1. Introduction

The plant pathogen *Claviceps purpurea* ((Fr.: Fr.) Tul.) is distributed worldwide and can infect more than 400 grass species including rye (*Secale cereale* L.) [1]. Germany, Poland, and Austria cover 73% of the European Union rye production of 4.6 million metric tons in 2018 [2]. Ergot is a severe fungal disease and the over-wintering bodies called sclerotia contain toxic ergot alkaloids (EAs). The EAs are a group of secondary metabolites [3] that are defined as derivatives of 4-(γ,γ-dimethylallyl)tryptophan (DMAT) [4]. EAs are known from several Ascomycetes species [5], including *C. purpurea* as a plant pathogen, and symbionts like *Neotyphodium* and *Epichloë* [6]. The ecological function is not fully clarified yet, but there are indications that EAs contribute to virulence [7] and play an important role for the resistance of the fungus against insects [6], mammals [8], or microbes [9]. More than 80 individual EAs are known in literature [5,10], which can be grouped into Clavine alkaloids, D-lysergic acid and its derivatives, and ergopeptines [4,11,12,13]. For *Claviceps* spp., the main EAs are ergometrine (Em), ergotamine (Et), ergosine (Es), ergocristine (Ecr), ergocryptin (Ekr), and ergocornine (Eco) along with their corresponding -inine epimers [14,15]. The pattern of the produced EAs strongly differs depending on fungal strain, host plant, and the geographic region [12,14,16,17]. Furthermore, a seasonal-dependent variation of the EA formation was observed for *Festuca sinensis* [18]. Additionally, for pathogenic variation, significant differences were observed among isolates according to their geographic origin [19]. Kodisch et al. [20] showed that isolates may also differ in their ergot severity on winter rye. For determination of EAs, numerous methods are reported in literature based on immunology, chromatography, or capillary electrophoresis [15,21,22,23]. For determination of individual EAs in food and feed at relevant levels, chromatographic methods such as high performance liquid chromatography with fluorescence detection (HPLC-FLD) and high performance liquid chromatography—tandem mass spectrometry (HPLC-MS/MS or LC-MS) are internationally validated [15,24] and were commonly used in several screening studies [1,17,25,26,27,28,29], especially when a high number of samples is needed to be screened in parallel [30]. Enzyme-linked Immunosorbent Assays (ELISA) are based on the antigen-antibody reaction and could be used to determine the content of a precursor in the ergot biosynthetic pathway to obtain information about the EA content. Additionally, ELISAs are easy-to-use, fast, and relatively cheap [31] and, therefore, the ideal solution for a quick and financially sustainable screening assay. Several commercial ELISA kits for evaluating EAs detecting lysergic acid and its derivatives as a common motif of one precursor group of the EAs are available [30,31,32]. Three commercially available ELISA kits have been shown for a small number of samples to screen EAs correctly in a qualitative manner in two cases, but not in a quantitative manner caused by false positive and false negative results when comparing to an HPLC approach [30]. The ELISAs show further cross reactivity against ergopeptines (e.g., ergovaline), which is not covered by the 12 EAs analyzed with the HPLC approach. This may lead to a higher total ergot alkaloid content by ELISA in comparison with the HPLC analysis [32].

Rye is especially prone to ergot contamination because it is a cross-pollinating crop opening the flowers wide and allowing the ergot spores to be brought directly into the pistils by aerosol deposition or insects [33]. Ergot infection is favored by cool, rainy weather when the flowers have a prolonged opening period, but also by a low amount of pollen shedding that is genetically inherited [20,34]. Ergot-contaminated rye grain was caused in the Middle Ages’ devastating epidemics in humans known as “St. Anthony’s fire” or ergotism with severe pathological syndromes such as gangrene, neurological diseases, and, finally, death [35,36]. In contrast, some EAs are used in medicine as therapeutic drugs to treat migraines, uterine hemorrhaging, or Parkinsonism [4] due to the pharmacological activities of these metabolites [12,36]. Since the second half of the 20th century, only single disease cases in humans are known and, currently, the risk of ergot as a fatal disease for humans and animals is low due to cleaning procedures, diversification of diets, and strict regulations of the ergot content within the European Union EU, [15,37]. Until now, the EU limits for ergot sclerotia and sclerotial fragments in unprocessed cereals are set to 0.05% by wt. for human consumption [38] and to 0.1% by wt. for animal feed [39]. However, reduction of the limit to 0.02% by wt. on unprocessed rye and maximum levels based on the EA alkaloid content as from 01.07.2022 onward to 250 µg/kg on rye milling products (until 30.06.2022: 500 µg/kg) and for infants and young children to 20 µg/kg are expected to be enacted for the 12 EAs ergometrine, ergometrinine, ergosine, ergosinine, ergotamine, ergotaminine, ergocornine, ergocorninine, α-ergocryptine, α-ergocryptinine, ergocrystine, and ergocrystinine (A. Raditschnig, pers. commun. 2020). However, not all milling companies are able to use photocell-based sorting machines because it enhances the costs and slows down the processing appreciably [33]. Furthermore, EAs could also be detected in ergot-cleaned grain samples likely caused by abrasion [40,41,42]. Gordon et al. [43] revealed that, in wheat and barley and, for a lesser extent, in rye, EAs also occurred in healthy grain that developed above and below the artificially infected individual florets. Therefore, it is not surprising that EAs occurred frequently in several studies in different countries and among all common cereals [44].

For rye and rye-based products, between 51% to 100% of all samples contained EAs with maximum levels ranging between 61 to 1231 µg/kg whereas, in one rye feeding lot, a maximum EA content of even 12,340 µg/kg was observed [1,17,25,26,27,28,29]. The maximum levels for other cereals such as wheat or triticale reached similar EA contents ranging between 123 and 1236 µg/kg [17,26,27,28,29,44], indicating the necessity of testing wheat and triticale for the presence of EAs. Furthermore, it is known that baking of wheat and rye flour reduces the EA content considerably [45,46] and also shifts the ratio between the epimeric forms toward the -inine forms [15]. However, this was not true for durum pasta production [47], so processing alone is not a guarantee to obtain alkaloid-free commodities. Although ergot often does not result in a large yield loss, ergot-contaminated grain can have a high economic impact on cereal production because of downgrading or rejecting grain lots contaminated with EAs [1,19,48]. The best measures for reducing ergot incidence and EA content is growing less susceptible cultivars, supporting the homogeneity of the rye stand, and controlling the weeds surrounding the fields [49]. However, up to now, it is not known whether physiological resistance exists in rye and breeding for lower susceptibility is difficult [33]. Only a few quantitative trait loci (QTL) were reported for assigning partial resistance to ergot, i.e., affecting the size and weight of sclerotia in bread wheat [50] or different components of the infection process like the hormonal pathway in durum wheat [51].

Regarding the recurring occurrence of EAs in rye and rye-based products, the future reduction of existing limits and expected maximum EU levels based on the EA content creates a need to clarify associated factors influencing EA content with the aim to develop and evaluate a quick screening method for farmers, small elevators, milling companies, and breeders. Otherwise, a high economic burden would arise for the industry, which likely would have an effect on the prize of the final products. Especially for selection for low EA content in breeding, cheap and quick tests are necessary because thousands of samples have to be screened in the short time frame between harvest and planting. The relationship of ergot severity and EA content is not fully clarified yet. Screening studies often consist of a low number of samples only and validation studies of HPLC and ELISA are rare in literature. Therefore, this correlation study was performed with a high number of samples to reveal (1) the covariation of ergot severity and EA content determined by HPLC in the light of different genotypes, environments (locations, years, countries), isolates, and infection levels and (2) the covariation between these traits and the commercial ErgoREAD ELISA test. For this, we analyzed 372 winter rye samples of several genotypes artificially infected by *C. purpurea* with three isolates at nine locations (Germany, Austria, Poland) in two years.

## 2. Results

### 2.1. Influence of Genotype, Environment, Isolate, and Interaction on Ergot Severity and EA Content

**Figure 1:**
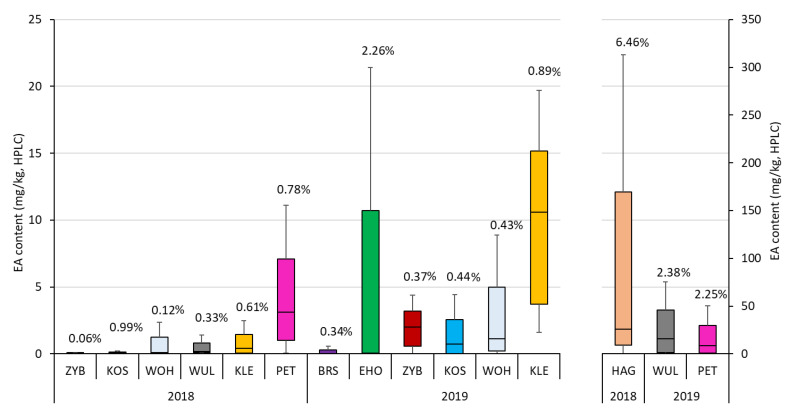
Boxplots of ergot alkaloids (EA) content determined by HPLC and mean ergot severity (% value above the respective column) of 15 environments for three winter rye genotypes (D.Amber, Elias, H_Hyb5) after inoculation with three isolates of *C. purpurea* of subset II (black line representing the median, error bars representing the standard error of means, WUL2019, PET2019, and HAG2018 are presented on a secondary y-axis due to their very high EA contents). For the abbreviations of the locations, please refer to Materials & Methods or Table S1.

The environments showed wide ranges of ergot severity and EA content determined by HPLC and ELISA, varying from 0.06% to 6.46%, 0 to 97.85 mg/kg, and 0.52 to 3673.89 mg/kg, respectively. Huge differences between the locations and years were observed concerning ergot severity and EA content determined by HPLC (Figure 1). The Austrian locations EHO 2018 and HAG 2018 showed the highest ergot severity, whereas the Polish location ZYB revealed low levels for ergot severity and EA content in both years.

**Figure 2:**
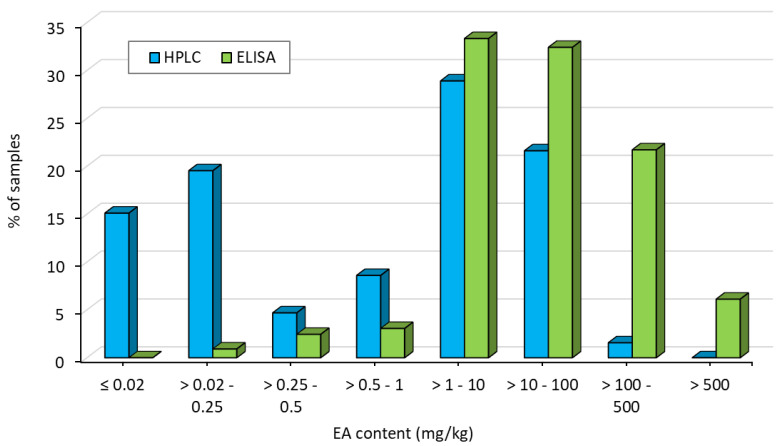
Distribution of ergot alkaloids (EA) content of subset I after inoculation with *C. purpurea* across 18 environments determined by HPLC (blue) and ErgoREAD ELISA (green, see Materials & Methods).

Small differences were observed for ergot severity among three rye genotypes with cultivar Elias showing significantly less ergot than H_Hyb5 (Table S2). Differences among the cultivars of the EA levels were not statistically significant as measured by both methods. ELISA resulted, on average, in a 43-fold higher EA content than HPLC. Similar large differences were obtained for the distribution of the EA content determined by HPLC and ELISA (Figure 2). For HPLC, most of the samples (40%) contained EA concentrations ≤0.25 mg/kg. In contrast, only 3% of the ELISA samples reached this low EA concentration level. Vice versa, the minority of the samples (2%) measured by HPLC contained higher EA levels than 100 mg/kg. For ELISA, however, 28% of the samples fell in this high EA class.

The environment was the most important component of variance for all three traits (Table S3). For ergot severity, genotype, isolate, and genotype × environment were also important sources of variance. There was no significant genotype × isolate interaction. For EA content measured by HPLC, isolate variance was also significant. No significant genotypic effect was determined for EA content, neither for HPLC nor for ELISA analyses. High entry-mean heritabilities were observed for ergot severity and EA content measured by HPLC (0.91 and 0.82, respectively). EA content measured by ELISA had a considerably lower heritability (0.57).

### 2.2. Covariation of Ergot Severity and Alkaloid Content Measured by HPLC

**Figure 3:**
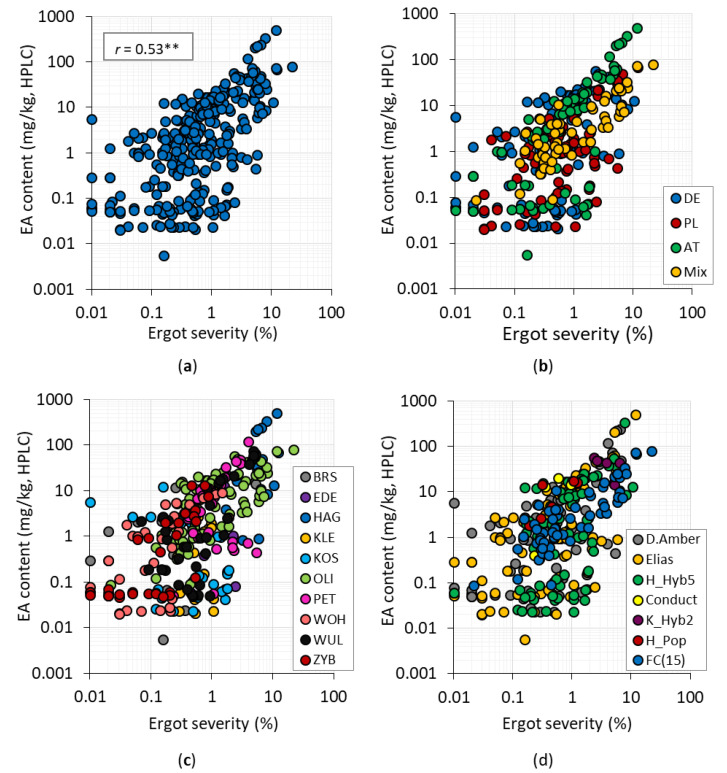
Correlation between ergot severity (%) and EA content determined by HPLC (mg/kg) of subset I after inoculation with *C. purpurea* (**a**) across all environments and isolates and colored by (**b**) origin of isolate (DE = German isolate, PL = Polish isolate, AT = Austrian isolate, Mix = Mix of all isolates [DE, PL, AT]), (**c**) location and (**d**) genotype (FC(15) = comprising 15 factorial crosses) (*r* = coefficient of correlation, **: significant at *p* < 0.01).

Ergot severity and EA content measured by HPLC showed good correlations of the respective two field plots (= replications) ranging from 0.70 to 0.91 (Figure S1) and illustrating that the genetic variation was high and the methods were reliable. A moderate positive covariation of ergot severity (%) and EA content determined by HPLC was obtained for subset I (*r* = 0.53, *p* < 0.01, Figure 3a) and subset II (*r* = 0.65, *p* < 0.01, data not shown). No grouping effects were observed when considering different factors such as isolate (Figure 3b), location (Figure 3c), and genotype (Figure 3d), i.e., the correlation did not improve. The correlations calculated within isolates (*n* = 96) and genotypes (*n* = 96) showed moderate to good correlations varying from *r* = 0.45 to *r* = 0.90. Splitting the samples in fractions of ergot severity <0.05% and ≥0.05% revealed low to medium covariations of *r* = 0.26 (*n* = 145) and *r* = 0.49 (*n* = 183), respectively.

### 2.3. Covariations with ErgoREAD ELISA Values

The ELISA showed a good correlation between the two field plots (= replications) for both ergot severity and EA content (Figure S1). No correlation, however, was detected between ergot severity (%) and EA content for subset I (*r* = −0.07) (Figure S2a) and subset II (*r* = −0.09) (data not shown). Comparing both analytical methods for determining EA content revealed no covariation between ELISA and HPLC for subset I (Figure S2b) and subset II (*r* = −0.04) (data not shown). No improvement of the correlation could be detected when splitting the dataset in the factors isolate, location, genotype, or infection level (ergot severity <0.05% and ≥0.05%).

### 2.4. Effect of the Isolates on Ergot Infection and EA Content

Across the years, significant differences between the isolates were observed for all traits except for ergot severity in 2018 and ergot severity across both years (Table 1). EA contents measured by ELISA were for all isolates substantially higher in 2019 than in 2018. This was, however, not the case for measurements by HPLC. The Austrian isolate always showed the highest EA contents measured by HPLC, which is significantly different from the Polish and German isolates. A table of ergot severity for the single rye genotypes separated by isolate and year could be found in the Supplementary Material (Table S4).

### 2.5. Composition of the EA Spectrum

**Figure 4:**
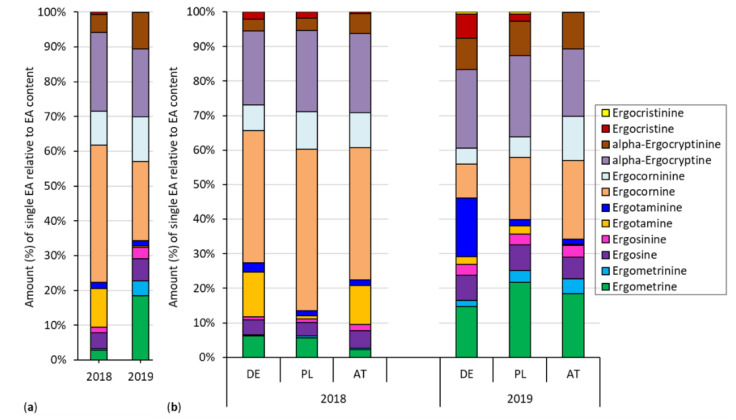
Amount (%) of single EAs relative to the EA content determined by HPLC after inoculation with *C. purpurea* of winter rye for 2018 and 2019 for (**a**): subset I and (**b**) subset II separated by three country-specific isolates (DE = German isolate, PL = Polish isolate, AT = Austrian isolate).

The pattern and distribution of all single EAs relative to the EA content determined by HPLC across all locations was mostly similar for both years (Figure 4a, subset I). Larger differences were found only for ergometrine and ergotamine. The amount of ergometrine was 6.3-fold higher in 2019 (19%) when compared to 2018 (3%), which is in contrast to the 30.6-fold decrease of ergotamine from 2018 (11%) to 2019 (0.36%). In almost all cases, the amount of the -inine forms of the respective EA was considerably lower than the -ine epimer, except for ergocristine (0.02%) and ergocristinine (0.11%) (2019, subset I) and for ergotamine (2%) and ergotaminine (17%) of the German isolate in 2019 (subset II), where the proportion of the -inine form was observed 8.5 fold higher than for ergotamine. Subset II showed a similar spectrum and ranking regarding the proportion across the three isolates (DE, PL, AT) and both years (2018, 2019) (Figure 4b). The EAs with the highest proportions were ergocornine and α-ergocryptine for both subsets except for the German isolate of 2019 in subset II. For subset II, α-ergocryptine followed by ergotaminine and ergometrine were detected as the most abundant EAs. Ergocristinine could be found only in traces. Ergocristine showed a higher amount in subset II than in subset I, especially for the German isolate in 2019 with a proportion of 7% on the EA content. A table with the content of all single EAs determined by HPLC of subset I after inoculation with *C. purpurea* from 2018 and 2019 could be found in the Supplementary Material (Table S5).

## 3. Discussion

For evaluating the covariation between ergot severity and EA content, artificial infection by spray inoculation was used. This is necessary to ensure a sufficiently high ergot infection and an even distribution across the experiment at all locations and years. Dung et al. [52] showed that natural ergot infection does not occur randomly within fields of Kentucky bluegrass (*Poa pratensis*) and that many factors, such as flowering, environmental or cultivation conditions, and occurrence of insects, contribute to disease proliferation. Nevertheless, Kodisch et al. [20] showed that ergot severity of genotypes after inoculation is highly correlated to that of natural infection. For our experimental set-up, it was previously described [34] that high heritabilities for ergot severity can be achieved. To achieve maximum variance necessary for calibration studies, we analyzed a dataset comprising nine locations in three countries, over two years, different rye genotypes, and three ergot isolates. We repeated each combination in the field experiment on two plots (replications).

### 3.1. Environment (Location × Year Combination) Has a High Impact

Ergot occurs naturally only sporadically and becomes a problem during wet and rainy weather conditions throughout the flowering stage [53]. Moist weather conditions around the time of meiosis have a negative effect on the production, viability, and movement of the pollen. Furthermore, honeydew becomes thick under warm and sunny conditions, which reduces ergot infections considerably [33]. Previous studies reported on a high influence of the environment on the ergot infection for sorghum (*Claviceps africana*) [54,55,56] and rye (*C. purpurea*) [20,34,57,58]. In our study, mean ergot severity showed a very large variation between environments ranging from 0.06% to 6.46% that might be caused by differences in weather despite artificial infection. These differences due to environments are highly significant, but clearly not predictable. The same location might have a low ergot severity in one year and a high severity in another year like WUL in our study (Figure 1). Therefore, we had highly differing ergot severities and EA contents according to the specific location × year combination. This explains, to some extent, the wide variation shown in Figure 1 and accords with the analysis of variance where the environment was the most important factor. The high genotype-by-environment interaction for ergot severity illustrates, once again, the necessity of multi-locational field trials [20,34,57,58]. One part of the environmental variation could be explained by the fact that ergot severity also depends on pollen shedding. Pollen shedding is affected by weather conditions as well [33]. With regard to the EA concentration, the mean ergot severity of an environment did not show any covariation with the mean level of EA concentration (Figure 1). For example, KOS 2019 and WOH 2019 nearly had the same ergot severity but differed in EA contents. On the other hand, the high EA contents of KLE 2019 or even PET and WUL 2019 are not mirrored in their ergot severity.

Ergot severity and EA content determined by HPLC had appreciable high heritabilities illustrating a strong genetic effect of these traits. EA contents determined by ELISA showed a moderate heritability only, which was likely caused by error-sensitive, adverse effects such as cross-reactivity or matrix effects. In conclusion, a clear influence of the environment affecting the ergot reaction and EA content measured by HPLC was detected.

### 3.2. Isolates Affect Ergot Severity and EA Formation in a Host-Unspecific Way

The isolates affected ergot severity and EA content measured by HPLC significantly, confirming earlier results [20]. The country-specific isolates in the balanced subset II in 2018 and across both years showed a similar ergot severity, but large differences concerning their EA quantity. Notably, the Austrian isolate produced a considerably higher EA quantity than the other isolates from Germany and Poland. In addition, Tittlemier et al. [59] showed that the isolate was a significant factor affecting EA concentrations in wheat sclerotia. Cagaš and Macháč [60] demonstrated for Kentucky bluegrass that isolates from different continents vary in their aggressiveness. Furthermore, Menzies et al. [19] showed a high pathogenic variation and significant differences among ergot isolates, according to the geographic origin in wheat. In this study, no significant genotype-by-isolate interaction was detected for all traits, i.e., the genotypes reacted similarly to the three isolates. Thus, the origin of the isolate must not be considered with a high effort in future testing systems, but the aggressiveness of the inoculum is crucial for an optimal differentiation of the genotypes. Additionally, EAs do not seem to act as major virulence factors in the infection process because an increased EA content of an isolate does not reflect a higher disease severity. In this context, studies have to be done to investigate the virulence impact of EAs in the biology of the fungal infection process, e.g., with EA defective *Claviceps* strains. In the literature, it is discussed that EAs are important for resistance of the fungus against insects [6], mammals [8], or microbes [9]. However, the ecological function of the EAs is not entirely clarified yet. Therefore, further approaches are needed to reveal the general role of EAs for the lifestyle of the fungus. Remarkably, all three country-specific isolates showed a similar EA spectrum.

### 3.3. EA Spectrum Was Rather Stable across Years and Isolates

EA spectrum was rather similar for isolates and years. In the majority of cases, the amount of the -inine epimers of the respective EA was clearly lower compared to the -ine epimer, as already demonstrated by Franzmann et al. [61]. Despite the -inine forms being mentioned to be biologically inactive, EAs are able to epimerize under various conditions (alkaline, acidic) [15]. The -inine forms can also re-interconvert in the primary form during processing [15]. In contrast, Diana Di Mavungu et al. [62] demonstrated that epimerization was minimal during the analytical process. Additionally, some EAs seems to be more stable regarding epimerization than others [63]. In our study, ergocristinine and ergotaminine showed higher amounts than the respective -ine epimers in a few cases. The mechanisms of epimerization are not fully clarified yet [63], but this shift towards the - inine forms might be caused by specific conditions (heat, solvent solution, light) during the growing season, processing, or analysis. Nevertheless, it is known that the main EAs as well as their -inine forms are considered in future regulations to be caused by their ability for epimerization [15].

In the EA spectrum, a shift of ergometrine and ergotamine in subset I occurred across the years. Notably, higher amounts for ergometrine were detected in 2019 than in the previous year and, for ergotamine, the opposite occurred. An explanation could be different environmental, climate, or nutritional conditions during the growing season, which possibly affect the formation of individual EAs. The higher values of the ELISA analysis indicate a higher diversity for the EAs and metabolites when compared to the HPLC. Ergocornine and α-ergocryptine were detected as prime EAs in our HPLC study, whereas ergocristinine could be found only in traces. In literature, the abundance of individual EAs varies with the experiment. According to European Food Safety Authority (EFSA) [15], ergotamine, ergocristine, ergosine, and ergocornine were generally found to be more abundant than α-ergocryptine and ergometrine. Additionally, Mulder et al. [27] detected α-ergocryptine as major EA, followed by ergosine, ergocornine, and ergotamine. In commercially available rye flour and rye products, ergocristine and ergotamine were found to be the major EAs [28,61,64]. In diverse cereal samples, ergosine occurred most frequently, while the highest levels were observed for ergotamine, ergocristine, or ergosine, depending on the product type [62]. In contrast, Blaney et al. [65] determined ergotamine as the most prominent EA in Australian rye and ergocristine was only present in very small amounts. It is well known that different isolates can produce different types of alkaloids [33]. However, in our study, all three isolates had a similar pattern. In *Epichloë*, gains and losses of EA-influencing genes led to chemotypic variation [13]. Thus, changes and shifting of the EA spectrum seem to occur regularly.

### 3.4. Covariation of Ergot Severity and EA Content is Moderate

The covariation between ergot severity and EA content will get a great importance when the EU limits for ergot in cereals will be based on EA concentrations because their determination by HPLC is costly and time consuming. Therefore, there would be a high economic extra load, if all samples are determined by this approach. A reasonably narrow correlation would allow us to identify grain lots with low ergot incidence and to concentrate the HPLC analyses on those samples with an EA level just above the limit. If this correlation is not useful, a rapid determination of EA concentrations by ELISA would be a good alternative. This technique could also be used by practical plant breeding companies to screen genotypes during the selection process. Therefore, we measured the EA content of our samples by both methods, the standard HPLC method, and one commercially available ELISA. HPLC analysis is a good and reliable tool for determining 12 EAs in a quantitative way [66], but the method needs a well-equipped laboratory with well-educated people [29,40]. In literature, ELISA approaches were already applied for evaluating EAs [67,68], but often with a lower number of samples and especially in the context of fescue toxicosis in livestock [69,70,71].

In our study, the results of the ErgoREAD ELISA analyses showed considerably higher EA contents for almost all samples when compared to HPLC with a notably high deviation in 2019. Accordingly, the distribution of the EA contents was completely different, as nearly all ELISA samples (92%) contained more than 1 mg/kg. Clearly, both methods measure EA concentrations in a completely different way. HPLC analysis determines the concentration of 12 main EAs, consisting of ergometrine, ergotamine, ergosine, ergocristine, α-ergocryptine, ergocornine, and the corresponding -inine epimers in a quantitative way [40]. In contrast, this ELISA is a qualitative method and measures lysergic acid as a progenitor in the EA pathway [32]. However, in the literature, >80 EAs are reported [5,10], so that, the individual 12 EAs analyzed by HPLC are only a subset of all existing EAs. Consequently, it is reasonable that the ELISA results overestimate the HPLC results. In this context, a varying consensus between both methods is also imaginable because the relative amount of the 12 analyzed EAs on all EAs can differ according to environmental conditions. Roberts et al. [70] already demonstrated that ergovaline concentrations determined by HPLC do not always correspond to the EA concentrations determined by ELISA. In addition, it is conceivable that matrix effects or cross-reactivity of components, which are comparable in structure falsify the results. Furthermore, the amount of cross-reactivity might vary between different groups of EAs [66] and usually ELISA approaches are less specific and accurate than HPLC methods [30]. However, all these factors cannot explain the 43-fold difference, on average, in EA concentrations between both methods (Table 1). 

A comparison of three commercially available ELISA kits [30] showed that two out of three ELISA tests considerably overestimated the EA contents in flour in most cases when following the original protocol. The highest amounts were often found with the ErgoREAD ELISA. In addition, only one kit was reliable in a narrow range from 100–500 µg/kg [30]. In our study, the ErgoREAD Elisa was performed according to the manufacturer recommendations. Furthermore, the extraction procedure and processing seem to have a high impact on the output and the working ranges of the commercially available ELISA kits that are often not large enough even for natural infections [30]. However, a high dilution factor as always necessary for samples from artificial inoculation adds an additional error.

The covariation between EA content measured either by HPLC or ErgoREAD ELISA was basically zero in our study (*r* = −0.04). Schnitzius et al. [71] also demonstrated that no consistent pattern between ELISA and HPLC existed for samples from Kentucky blue grass. This indicates a discrepancy in the analytical detection due to a higher cross reactivity in the ELISA in comparison to the limited detection of EAs in the HPLC. Splitting the dataset into the factors isolate, environment, genotype, or infection level (ergot severity <0.05% and ≥0.05%) revealed no better relationship between both methods. A cause could be the different spectrum of chemical compounds analyzed by both methods. The HPLC focuses on the 12 selected EAs and cannot detect lysergic acid based on precursors and metabolites, which are not covered by the HPLC, as it is the case for ergopetides like ergovaline or higher protein conjugates, which could occur in nature. However, previous studies showed in *C. purpurea* that ergovalines seem to occur only in very low quantities [15]. Using the ELISA for a rapid screening of the EAs might give a higher security not to underestimate the EA concentration in a sample.

The samples in this approach were assembled by considering relevant criteria such as genotypes, environments (locations, years, country), and isolates to receive a maximal variation regarding ergot severity and EA content. This is the usual procedure for calibration studies [66,72,73]. This study revealed only a moderate positive covariation between ergot severity and EA content determined by HPLC for both subsets, as previously demonstrated [27,69,74,75,76]. In our study, a large deviation from the regression reduced the coefficient of correlation to *r* = 0.53 (Figure S2a). A cause could be that, even in sclerotia of similar weight, the total amount of ergot alkaloids varied significantly [75]. The deviation is especially large for low ergot severities. For example, an ergot severity of 0.16% showed EA contents from 0.006 to 12.33 mg/kg. Conversely, with an EA content of 0.5 mg/kg, ergot severities varied from 0.01% to 9%. That implies that, for low EA concentrations, the prediction of the EA content based on the amount of ergot in grain is even more precarious, likely because the coefficient of (error) variation increases with declining EA concentrations [75]. Low EA concentrations, however, correspond even more to a practical situation than our study because screening studies of naturally infected rye and rye-derived products normally contain lower EA concentrations than those with artificial infection [1,17,26,27,28,29,36,77]. However, the grouping of samples after infection level (ergot severity <0.05% and ≥0.05%) did not improve the covariation in our study. The same is true for differentiating the samples according to genotype, environment, or isolate. Some locations showed high correlations, but this was often not stable across the years and is, therefore, not predictable for a single location. Although the Austrian isolate showed a high correlation for both years, this was not consistent for all other isolates. Therefore, a prognosis of the EA content based on the ergot amount is not possible because, in nature, there are always several isolates appearing.

In consequence, this study indicates that the EA content cannot be predicted in a reliable way based on ergot severity. Mulder et al. [27] and Schummer et al. [78] came to the same conclusion when analyzing a lower number of ergot samples from various cereals.

## 4. Conclusions

The isolates showed rather stable EA profiles even though they were obtained from three countries. There were slight differences among years. We got visible ergot infections in all environments. The ergot severity, however, cannot predict the EA concentrations in a given sample as measured by HPLC. This was even more clear for low ergot severities that are usually occurring in natural infections. Clearly, the EA contents have no relationship to disease severity and their absolute levels are not an important factor of the ergot infection in winter rye. Moreover, contents of individual EAs are most likely governed by different weather or physiological conditions than by ergot infection since only a moderate covariation could be found despite both traits having a similar high heritability when EA contents were measured by HPLC. To fulfill the future EU limits of 250 µg/kg for human consumption (except for baby food), HPLC analyses are necessary at least for those rye lots that visibly show some sclerotia. Many with a high sclerotia incidence will be rejected right away. However, even without visible infection in a sample, EAs might be present and may be caused by relocation within the ear or dust and abrasion from a few sclerotia during harvesting. The used ELISA did neither show a correlation to ergot severity nor to the EA concentrations measured by HPLC confirming previous findings. Further research is needed (1) to understand the factors influencing ergot infection more precisely as well as EA formation and their relationship and (2) to obtain a quick, cheap, and easy-to-use screening assay for the practical and commercial use. At the moment, the only recommendation could be for human consumption to concentrate on samples with minimal percentages of ergot sclerotia by visual means and to analyze them for their EA content by HPLC methods to meet the EU regulations.

## 5. Materials and Methods

### 5.1. Samples, Field Trials, and Inoculation Procedure

In total, 372 winter rye samples were used for this correlation study. The examined samples consisted of a diverse set of genotypes, locations, years, and isolates (Table 2). The following declaration is implemented in the study. Subset I consisted of all 372 winter rye samples and subset II consisted of 288 winter rye samples orthogonally distributed across genotypes, isolates, and environments (location-year combinations). The experiment was performed in 2018 and 2019 at the following 10 locations: Oberer Lindenhof (OLI), Braunschweig (BRS), Wohlde (WOH), Petkus (PET), Wulfsode (WUL), and Kleptow (KLE) in Germany, Zwettl-Edelhof (EHO), and Hagenberg (HAG) in Austria, Kościelna Wieś (KOS), and Zybiszów (ZYB) in Poland. BRS could not be analyzed in 2018 due to missing infections, and HAG in 2019 due to severe hail destroying the crop shortly before harvest. A detailed list of locations and their characteristics can be found in the Supplementary Material (Table S1). The experiment was completely randomized with two replicates in a chessboard-like design. Therefore, each entry plot was surrounded by four plots of triticale (×Triticosecale Wittm.) as “border plots” [34]. Depending on the location, the size of the large-drilled plots ranged from 5.0 to 7.04 m^2^. The varieties were supplied by the respective breeding companies (Table 2). Local triticale cultivars were grown as border plots.

Genotypes were inoculated with each of three inocula collected from sclerotia of infected rye in Germany (DE), Poland (PL), and Austria (AT) at nine locations in 2018 and 2019, comprising subset II. They were planted as split-plot design (main plot = isolate, subplot = genotype). Additionally, six other genotypes were inoculated at OLI in both years with the German isolate only and 15 factorial crosses (FC(15)) largely differing for their amount of pollen shedding with a mix of all three isolates (Table 2).

Seed density amounted to about 200 kernels/m^2^ and sowing was done from mid-September to early October. Mineral fertilizers, herbicides, growth regulators, and fungicides were applied at each location in a conventional way. For inoculum production for both years of field testing, each country sent ergot samples in 2017 to the lab of B. Rodemann to ensure that the same inoculum was used for both years. The ergot samples were collected from rye stands from the main growing areas. As previously described in detail by Miedaner et al. [79], all inocula were produced for all location sites and years by Julius Kühn-Institute, Institute for Plant Protection in Field Crops and Grassland (Braunschweig, Germany) (JKI) by isolating *Claviceps purpurea*, according to Kirchhoff [80] and producing conidia suspension for inoculation on wheat-grain medium, according to Mielke [81] and Engelke [82]. Inoculation was started when the earliest 30% of the plots were fully flowering (BBCH 65, [83]) via spraying with a machine-driven field sprayer in the evening (5:30–9 p.m.) or in the morning (8–10:30 a.m.), repeated up to five times within a one-day to four-day interval, according to the temperature. The aim was that all plots were inoculated at least once at their respective flowering time. Harvesting was done by cutting a 1-m^2^ subplot from the middle of the large plot by hand at the dough ripening stage (BBCH 85–89) using only primary tillers. Afterward, all heads of one plot were air dried (30 °C) and threshed by a large single-head thresher (Pelz K 35, Saatzuchtbedarf Baumann, Waldenburg, Germany).

### 5.2. Sample Preparation

All sclerotia fragments were sorted out by hand or by an optical sorting machine (SATAKE Pikasen FMS 2000 F (2017/18)/ ANYSORT G64 #G64A-G112 (2018/19), Ruttmann, Hamburg, Germany) depending on the cooperation partner and the components (grain, ergot) were weighed to calculate ergot severity as a percentage of sclerotia relative to the total grain sample by weight. Afterward, a sub-sample of 200 g of grain and sclerotia were merged regarding the original ergot severity. This procedure was necessary because the samples from field plots were too large to handle. The 200-g sub-samples were milled (Ultra Centrifugal Mill ZM 200, 1 mm sieve, Retsch, Haan, Germany) and the flour was divided for HPLC and ELISA analyses.

### 5.3. High Performance Liquid Chromatography (HPLC) Analysis of EAs

HPLC analysis was conducted by the Austrian Agency for Health and Food Safety, Institute for Food Safety Linz, (AGES, Linz, Austria). Wet-chemical extraction, purification, and HPLC analysis were done according to BVL L 15.01/02-5:2012-01 [84] with some modifications. Briefly, EAs of 20 g flour were extracted with 100 mL of extraction solvent consisting of ethyl acetate (C_4_H_8_O_2_)/methanol (CH_3_OH)/ammonia (NH_3_, 25%)/isopropyl alcohol (C_3_H_8_O) (75/5/7/7 (*v*/*v*/*v*/*v*)). Purifying of the extract with a solid-phase column (Alumina B) is followed by concentrating. EAs were separated using a Zorbax Eclipse XDB-C_18_-HPLC-column (Agilent, Santa Clara, CA, USA) (Flow: 1 mL/min, Eluent: acetonitrile (C_2_H_3_N)/ ammonium carbamate (CH_6_N_2_O_2_) solution 0.2 g/L, 50/50 (*v*/*v*)) and detected via Fluorescence detection (excitation λEx: 330 nm, emission λEm 415 nm). Calculating EA content was done by 3-point calibration and including multipliers according to the processing steps in the lab. The examined 12 EAs were: ergometrine, ergometrinine, ergosine, ergosinine, ergotamine, ergotaminine, ergocornine, ergocorninine, α-ergocryptine, α-ergocryptinine, ergocrystine, and ergocrystinine. For calculating the EA content, all individual EAs were summed up. In the following, the terms “ergot alkaloid content (determined) by HPLC” refer to the sum of these 12 EAs. All individual EA values below the limit of quantitation (LOQ < 0.02 mg/kg) were considered as zero. EHO 2018 showed no detectable level of EAs even though all samples were independently analyzed two times and was, therefore, not included in the respective analyses.

### 5.4. Enzyme-Linked Immunosorbent Assay (ELISA) of EAs

ErgoREAD ELISA, known as a competitive ELISA for detection of EAs in wheat, rye, and triticale samples, was purchased from LCTech GmbH (Daimlerstraße 4, Obertaufkirchen, Germany) and the analysis was performed at UHOH. A wet-chemical extraction of the EAs was carried out by mixing 20 g of the flour with 50 mL methanol (CH_3_OH)/ phosphoric acid (H_3_PO_4_, 0.25%) (40/60 (*v*/*v*)) and shaking for 20 min (165 rpm, Orbital Shaker 3017, GFL Gesellschaft für Labortechnik GmbH, Burgwedel, Germany). The extract was filtered by rinsing through glass funnels with filter papers (Qualitative circles, 150 mm Ø, Cat No 1001 150, Whatman, Maidstone, United Kingdom). After dilution, the samples were filtered again with a syringe filter (pore size: 0.45 µmm, ø 15 mm, unster., PTFE, Rotilabo^®^, Roth, Karlsruhe, Germany) to remove all suspended solids. This was followed by proceeding the protocol of the ELISA Kit, measuring the extinction values by a microplate reader (Sunrise, Tecan Group Ltd., Männedorf, Switzerland) using the integrated Magellan software (Tecan Group Ltd., Männedorf, Switzerland) relative to the standard samples (0 ppb, 0.025 ppb, 0.1 ppb, 0.25 ppb, 0.5 ppb, 0.75 ppb, 1 ppb), covering the range from 0 to 5 ppm after taking the sample dilution factor into account. Higher concentrations of EAs could be analyzed by further sample dilution. Calculating the EA content was based on the company-owned software of LCTech GmbH (Obertaufkirchen, Germany). All samples were analyzed in duplicate.

### 5.5. Statistical Analyses

Single-plot data were used as a basis for all analyses. According to Bernal-Vasquez et al. [85], outlier tests were performed and detected outliers were considered as missing values. For conducting analyses of variance (ANOVA), a square-root transformation was done for all traits because the residuals were not normally distributed in any environment for biological reasons. ANOVA was first done for each location separately and, second, combined across locations for each trait using standard procedures [86]. For all ANOVAs, the effect of the factors ‘genotype’ and ‘isolate’ were considered as fixed and the factors ‘replication’ and ‘environment’ as random. A significance level of 0.05 or 0.01 was applied to all statistical calculations. The estimates of the ANOVA of the ratio of genotypic to phenotypic variance considering the number of replicates were used to calculate repeatability for each environment and entry-mean heritability (*H^2^*) across all environments considering the number of replicates and environments, respectively [87]. The software packages R [88] and R-Studio (Version 3.5.1) [89] were used to perform the outlier test and ANOVAs for calculating the means and graphic visualization. The overall means reported in the tables were back-transformed. In the figures, the original means are reported. Multiple testing was conducted by a Tukey test as implemented in the R-studio.

## Figures and Tables

**Table 1:** Means and significance level of ergot severity and EA content determined by HPLC or ELISA across up to eight locations in two years and across years after inoculation with *C. purpurea* with three country-specific isolates (DE = German isolate, PL = Polish isolate, AT = Austrian isolate) for subset II.

Trait	2018			2019			2018 + 2019		
Isolate	DE	PL	AT	DE	PL	AT	DE	PL	AT
**Ergot Severity (%)**	2.04a ^1^	2.07a	1.53a	0.67a	1.13b	1.72c	1.36a	1.60a	1.62a
**EAs, HPLC (mg/kg)**	1.10a	6.21a	37.64b	8.41b	0.80a	16.75c	5.00a	3.32a	26.50b
**EAs, ELISA (mg/kg)**	7.5a	26.8b	23.5b	862.9ab	1700.7a	254.8b	387.7a	824.8b	120.7a

^1^ Treatments with the same letter within one row are not significantly different (Tukey test, *p* < 0.05).

**Table 2:** Genotype, breeding company, test environments (= location × year combinations), and inoculated isolate(s) of all 372 winter rye samples (= subset I) and the orthogonal samples (= subset II); FC(15) = 15 factorial crosses, DE = German isolate, PL = Polish isolate, AT = Austrian isolate, Mix = Mix of all isolates (DE, PL, AT).

Genotype	Breeding Company	Test Environments		Isolate(s)	No. of Samples
		**Number**	**Name**		
D.Amber ^1^	“DANKO” Hodowla Roslin Sp. z o.o.	16	All ^2^	DE, PL, AT	96
Elias ^1^	Saatzucht LFS Edelhof	16	All ^2^	DE, PL, AT	96
H_Hyb5 ^1^	HYBRO Saatzucht GmbH & Co. KG	16	All ^2^	DE, PL, AT	96
D.Amber	“DANKO” Hodowla Roslin Sp. z o.o.	2	OLI	DE	4
Elias	Saatzucht LFS Edelhof	2	OLI	DE	4
H_Hyb5	HYBRO Saatzucht GmbH & Co. KG	2	OLI	DE	4
Conduct	KWS LOCHOW GmbH	2	OLI	DE	4
K_Hyb2	KWS LOCHOW GmbH	2	OLI	DE	4
H_Pop	HYBRO Saatzucht GmbH & Co. KG	2	OLI	DE	4
FC(15)	KWS LOCHOW GmbH	2	OLI	Mix	60

^1^ comprising subset II. ^2^ except BRS 2018, HAG 2019, OLI 2018, 2019.

## Supplementary Materials

The following are available online at https://www.mdpi.com/2072-6651/12/11/676/s1. Figure S1: Correlation of the replications (repeatability) of subset I of (a) ergot severity (%), and EAs measured with (b) HPLC, and (c) ELISA. Figure S2: Correlation of subset I after inoculation with *C. purpurea* across 18 environments between (a) ergot severity (%) and EA content determined by ELISA (mg/kg) and (b) EA content determined by HPLC and ELISA (mg/kg) (*r* = coefficient of correlation). Table S1: Location site, country, abbreviation, and characteristics of all field trials. Table S2: Means, ranges (in brackets), and least significant difference (LSD5%) for ergot severity (%), EAs (mg/kg) determined by HPLC, and ELISA for each genotype after inoculation with *C. purpurea* across 15 environments in subset II. Table S3: Estimates of variance components and entry-mean heritabilities for ergot severity (sq transf. = square root transformed) and EA contents determined by HPLC and ELISA after inoculation with *C. purpurea* across 15 environments in subset II. Table S4: Mean for ergot severity (%) and EAs (mg/kg) determined by HPLC and ELISA of subset II for each genotype and country-specific isolate (DE = German isolate, PL = Polish isolate, AT = Austrian isolate) across eight locations in 2018 and 2019. Table S5: Content (mg/kg, average) of single EAs determined with HPLC of subset I after inoculation with *C. purpurea* for 2018 and 2019.
