# Supplementary material for: Covariation of Ergot Severity and Alkaloid Content Measured by HPLC and One ELISA Method in Inoculated Winter Rye across Three Isolates and Three European Countries
Source: Toxins (Basel). 2020 Oct 26;12(11):676. doi: 10.3390/toxins12110676 (PMC7692364; doi:10.3390/toxins12110676)
Supplement: Supplementary file 1 [file toxins-12-00676-s001.pdf]

Supplementary Materials: Covariation of Ergot Severity and Alkaloid Content Measured by HPLC and One ELISA Method in Inoculated Winter Rye across Three Isolates and Three European Countries

Anna Kodisch, Michael Oberforster, Armin Raditschnig, Bernd Rodemann, Anna Tratwal, Jakub Danielewicz, Marek Korbas, Brigitta Schmiedchen, Jakob Eifler, Andres Gordillo, Dörthe Siekmann, Franz Joachim Fromme, Frederik N. Wuppermann, Franz Wieser, Elisabeth Zechner, Małgorzata Niewińska and Thomas Miedaner

Table S1. Location site, country, abbreviation and characteristics of all field trials.

Location Site	Country	Abbreviation	Soil Type	Mean Annual Temperature [°C]	Mean Precipitation [mm]
Ob. Lindenhof (48°28'25.5"N 9°18'17.9"E)	Germany	OLI	Brown soil	6.6	952
Braunschweig (52°16'33.4"N 10°34'09.3"E)	Germany	BRS	Sandy loam	9.3	570
Wohlde (52°48'48.7"N 9°59'53.1"E)	Germany	WOH	Sand	9.3	950
Petkus (51°58'50.3"N 13°21'01.7"E)	Germany	PET	Sand	8.5	596
Wulfsode (53°03'45.6"N 10°14'02.5"E)	Germany	WUL	Sand	9.3	852
Kleptow (53°21'54.9"N 14°00'04.8"E)	Germany	KLE	Loamy Sand	9.6	475
Zwettl-Edelhof (48°36'23.5"N 15°13'13.3"E)	Austria	EHO	Brown soil	7.7	657
Hagenberg (48°22'28.4"N 14°30'51.2"E)	Austria	HAG	Brown soil	9.1	772
Kościelna Wieś (51°46'28.7"N 18°00'58.0"E)	Poland	KOS	Brown soil	8.6	510
Zybiszów (51°03'51.9"N 16°54'45.4"E)	Poland	ZYB	Degraded black soil	9.1	571

Table S2. Means, ranges (in brackets) and least significant difference (LSD5%) for ergot severity (%), EAs (mg/kg) determined by HPLC and ELISA for each genotype after inoculation with Claviceps purpurea across 15 environments in subset II.

Genotype	Ergot Severity (%)	EAs, HPLC (mg/kg)	EAs, ELISA (mg/kg)
**H_Hyb5**	1.78 (0.01–9.3) a[1]	10.50 (0–337.13) a	473.9 (0.2–13,728.7) a
**D.Amber**	1.47 (0.02–10.5) ab	11.41 (0–239.32) a	441.2 (0.4–7595.9) a
**Elias**	1.32 (0.02–8.48) b	12.92 (0–498.40) a	494.5 (0.3–5387.6) a
**Mean**	1.53	11.61	468.9
**LSD5%**	0.32	7.44	218.8

[1] Treatments with the same letter are not significantly different (Tukey test, $p < 0.05$)

Table S3. Estimates of variance components and entry-mean heritabilities for ergot severity (sq transf. = square root transformed) and EA contents determined by HPLC and ELISA after inoculation with Claviceps purpurea across 15 environments in subset II.

Parameter	Degrees of Freedom	Ergot Severity (%) (sq Transf.)	HPLC (mg/kg) (sq Transf.)	ELISA (mg/kg) (sq Transf.)
Variance components: Environment (E)	14	6.122 **	67.04 **	3051.2 **
Genotype (G)	2	1.615 **	0.21	14.5
Isolate (I)	2	2.012 **	83.01 **	322.6
G × E	28	0.139 **	0.97	70.1
G × I	4	0.031	2.56	96.6
G × E × I	56	0.040	2.02	217.2 *
Error	120	0.037	1.57	130.9
Heritability		0.92	0.82	0.57

*, **: significant at $p < 0.1$, $p < 0.05$ and $p < 0.01$, respectively

Table S4. Mean for ergot severity (%) and EAs (mg/kg) determined by HPLC and ELISA of subset II for each 48 genotype and country-specific isolate (DE = German isolate, PL = Polish isolate, AT = Austrian isolate) across 8 49 locations in 2018 and 2019.

Trait	Year	Isolate	D.Amber	Elias	H_Hyb550
**Ergot severity (%)**	2018	DE	2.14	1.71	51<br>2.27
		PL	2.17	1.70	2.3452
		AT	1.20	1.63	1.75
	2019	DE	0.59	0.57	0.8553
		PL	1.16	0.84	1.39 54
		AT	1.58	1.49	2.09
**HPLC (mg/kg)**	2018	DE	1.23	0.43	1.6555
		PL	5.71	4.50	8.43
		AT	33.96	52.20	56<br>26.76
	2019	DE	7.43	6.33	11.4657
		PL	0.75	0.84	0.80 58
		AT	20.19	15.51	14.55
**ELISA (mg/kg)**	2018	DE	6.94	3.28	11.9859
		PL	26.66	20.84	32.51
		AT	23.77	27.96	60<br>18.68
	2019	DE	1195.29	1064.69	317.8861
		PL	1433.75	1490.36	2114.36
		AT	113.27	539.17	62<br>176.55

Table S5. Content (mg/kg, avarage) of single EAs determined with HPLC of subset I after inoculation with 66 Claviceps purpurea for 2018 and 2019.

Single EAs	Content (HPLC, mg/kg)	
	**2018**	**2019**
**Ergometrine**	538.93	1659.09
**Ergometrinine**	88.18	308.06
**Ergosine**	717.14	712.09
**Ergosinine**	227.05	341.01
**Ergotamine**	1916.17	133.38
**Ergotaminine**	300.92	572.89
**Ergocornine**	4783.26	1609.53
**Ergocorninine**	1192.67	821.94
**alpha-Ergocryptine**	2874.44	2367.83
**alpha-Ergocryptinine**	680.90	975.78
**Ergocristine**	1170.44	262.17
**Ergocristinine**	226.69	45.80

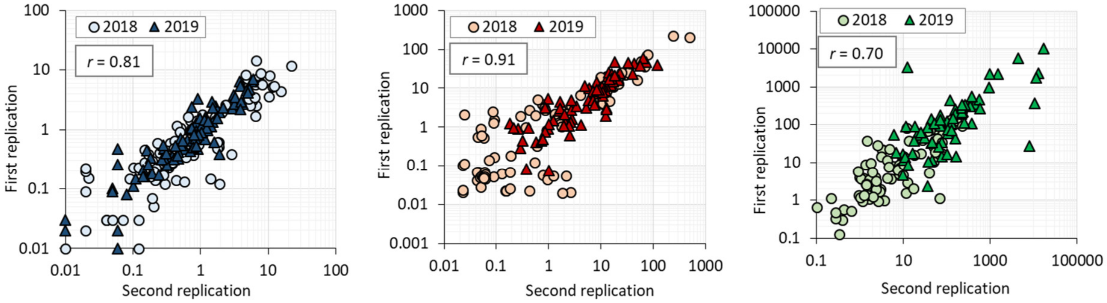

(**a**) Ergot severity (%) (**b**) EAs (mg/kg) determined by HPLC (**c**) EAs (mg/kg) determined by ELISA

Figure S1. Correlation of the replications (repeatability) of subset I of (**a**) ergot severity (%), and EAs (mg/kg) determined by (**b**) HPLC, and (**c**) ELISA.

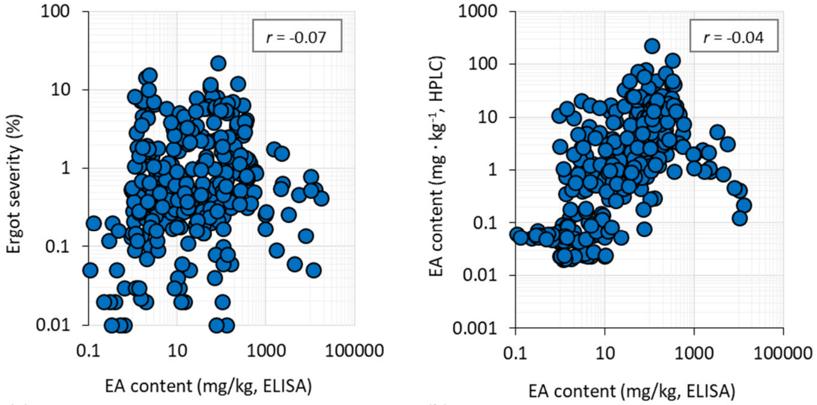

(**a**) (**b**)

Figure S2. Correlation of subset I after inoculation with *Claviceps purpurea* across 18 environments between (**a**) ergot severity (%) and EA content determined by ELISA (mg/kg) and (**b**) EA content determined by HPLC and ELISA (mg /kg) (r = coefficient of correlation).
